# Prevalence of Atherosclerosis in diabetic and non-diabetic patients with rheumatoid arthritis

**DOI:** 10.12669/pjms.315.7620

**Published:** 2015

**Authors:** Bartłomiej Kisiel, Robert Kruszewski, Aleksandra Juszkiewicz, Krzysztof Kłos, Małgorzata Tłustochowicz, Witold Tłustochowicz

**Affiliations:** 1 Bartłomiej Kisiel, MD, PhD, Department of Internal Diseases and Rheumatology, Military Institute of Medicine, Warszawa, Poland; 2 Robert Kruszewski, MD, Department of Internal Diseases and Rheumatology, Military Institute of Medicine, Warszawa, Poland; 3 Aleksandra Juszkiewicz, MD, PhD. Department of Internal Diseases and Rheumatology, Military Institute of Medicine, Warszawa, Poland; 4 Krzysztof Kłos, MD, PhD. Department of Infectious Diseases and Allergology, Military Institute of Medicine, Warszawa, Poland; 5 Małgorzata Tłustochowicz, MD, PhD. Department of Internal Diseases and Rheumatology, Military Institute of Medicine, Warszawa, Poland; 6 Witold Tłustochowicz, MD, PhD. Department of Internal Diseases and Rheumatology, Military Institute of Medicine, Warszawa, Poland

**Keywords:** Atherosclerosis, Diabetes mellitus, Rheumatoid arthritis

## Abstract

**Objectives:**

(1) To compare the prevalence of preclinical atherosclerosis in diabetic vs. non-diabetic rheumatoid arthritis (RA) patients; (2) to determine the influence of classical and RA-related factors on atherosclerosis; (3) to assess the usefulness of combined carotid and femoral ultrasonography in detecting atherosclerosis.

**Methods:**

The study comprised 42 non-diabetic RA patients, 42 diabetic RA patients and 42 controls. Intima media thickness (IMT) was measured in the common carotid and superficial femoral arteries. These vessels were screened for atherosclerotic plaque.

**Results:**

Plaque was more prevalent in diabetic RA patients than in non-diabetic RA patients or controls. Carotid IMT and femoral IMT were higher in diabetic RA patients compared to controls. So was femoral IMT in diabetic compared to non-diabetic RA patients. The prevalence of increased IMT and plaque was comparable in carotid ultrasonography and combined carotid and femoral ultrasonography in all groups.

**Conclusions:**

Subclinical atherosclerosis was found to be higher in diabetic RA patients than in non-diabetic RA patients. The combination of carotid and femoral artery ultrasonography did not improve the detection of atherosclerosis in RA.

## INTRODUCTION

Rheumatoid arthritis (RA) and diabetes mellitus (DM) are associated with increased cardiovascular (CV) morbidity and mortality. Several studies have suggested that RA equals DM as a risk factor for CV disease.^1^-^3^

Several studies have assessed the prevalence of DM in RA patients but the results are contradictory. Han et al. reported a higher prevalence of type 2 DM in RA patients compared to age- and sex-matched controls,^4^ but other studies found no relationship between RA and DM.^5^,^6^ However, it should be emphasized that abnormalities in glucose metabolism (i.e. insulin resistance- IR) have been well documented in RA and may correlate with RA activity.^7^ Similarly to what is observed in general population, IR seems to be an independent CV risk factor in RA patients.^8^,^9^ Thus, one might wonder whether overt DM increases CV risk in RA patients substantially or it is moderate because of abnormalities in glucose metabolism, which exists in many RA patients.

Very few studies have compared atherosclerosis in non-diabetic RA patients with that in RA patients with abnormalities in glucose metabolism. Dessein et al. found that in RA metabolic syndrome (MS) and IR were associated with the carotid intima media thickness (CIMT).^10^ Chung et al. reported an association between MS and a higher coronary-artery calcification score.^8^

Several non-invasive diagnostic tools, such as measuring CIMT and carotid plaque may be used to detect subclinical atherosclerosis. Most studies assessing the IMT and plaque have focused on carotid arteries. However, some studies have suggested that combined evaluation of the carotid and femoral arteries may be better in assessing atherosclerotic burden than the evaluation of a single vascular bed. Li et al. found that combination of carotid and femoral ultrasound can significantly improve the detection of atherosclerosis in type 2 diabetes.^11^

The aims of the study were (i) to assess preclinical atherosclerosis in diabetic and non-diabetic RA patients; (ii) to determine the influence of classical and RA-related factors on atherosclerosis; (iii) to determine if a combined ultrasonography of carotid and femoral arteries is superior to carotid ultrasonography only in detecting preclinical atherosclerosis in RA.

## METHODS

The study was approved by the Ethics Committee of the Military Institute of Medicine, Warsaw, Poland. Each participant signed an informed consent form.

### Patients

The study comprised 42 non-diabetic RA patients (NDRA), 42 diabetic RA patients (DRA) and 42 age- and sex-matched healthy individuals. Patients with clinically overt coronary artery disease or history of stroke were excluded. A complete history, physical examination and laboratory tests were performed and recorded in a standard protocol (Table-I, Table-II). Hands and feet X-rays were performed in 67 RA patients. RA activity was assessed with disease activity score in 28 joints (DAS28). Framingham 10-year risk score (FSS) was used to assess CV risk related to classical risk factors.

**Table-I:** Study and control groups characteristics.

	Controls (n=42)	NDRA (n=42)	DRA (n=42)	P value	Post-hoc analysis
Age, years	63.31 (8.07)	63.95 (10.77)	63.95 (10.84)	0.94	n/a
Males	6 (14.29%)	6 (14.29%)	6 (14.29%)	1.0	n/a
Ever-smokers	17 (40.48%)	18 (42.86%)	18 (42.86%)	0.75	n/a
Pack-years	10.08 (15.19)	12.81 (20.77)	7.81 (11.76)	0.38	n/a
BMI, kg/m^2^	27.49 (3.94)	26.87 (4.20)	28.31 (4.44)	0.30	n/a
Hypertension	21 (50.00%)	30 (71.43%)	35 (83.33%)	0.004	Controls vs NDRA: *P*=0.044
					Controls vs DRA: *P*=0.0012
					NDRA vs DRA: *P*=0.19
ESR, mm/h	11.32 (12.18)	35.60 (22.70)	38.88 (29.67)	<1×10^-6^	Controls vs NDRA: *P*=4×10^-6^
					Controls vs DRA: *P*<1×10^-6^
					NDRA vs DRA: *P*=0.51
CRP, mg/dL	0.51 (1.01)	2.63 (3.37)	2.71 (3.60)	0.0009	Controls vs NDRA: *P*=0.0013
					Controls vs DRA: *P*=0.0008
					NDRA vs DRA: *P*=0.90
Total cholesterol, mg/dL	218.97 (47.29)	196.50 (36.87)	197.36 (46.15)	0.033	Controls vs NDRA: *P*=0.022
					Controls vs DRA: *P*=0.025
					NDRA vs DRA: *P*=0.93
LDL cholesterol, mg/dL	131.55 (41.12)	113.03 (35.61)	111.86 (38.09)	0.04	Controls vs NDRA: *P*=0.035
					Controls vs DRA: *P*=0.025
					NDRA vs DRA: *P*=0.90
HDL cholesterol, mg/dL	61.14 (15.10)	61.13 (17.67)	52.57 (14.10)	0.026	Controls vs NDRA: *P*=0.99
					Controls vs DRA: *P*=0.017
					NDRA vs DRA: *P*=0.02
Triglycerides, mg/dL	135.57 (71.93)	122.70 (52.73)	158.16 (76.57)	0.08	n/a
Framingham 10-year risk score	9.80 (3.99)	8.26 (4.98)	16.54 (8.05)	<1×10^-6^	Controls vs NDRA: *P*=0.25
					Controls vs DRA: *P*=2×10^-6^
					NDRA vs DRA: *P*<1×10^-6^

Data is presented as mean (standard deviation) for continuous variables and number (percentage) for categorical variables. BMI- body mass index. CRP- C reactive protein. DRA- diabetic RA patients. ESR- erythrocyte sedimentation rate. NDRA- non-diabetic RA patients.

**Table-II:** Study groups characteristics.

	NDRA (n=42)	DRA (n=42)	P value
Disease duration, years	11.81 (10.44)	12.83 (8.67)	0.63
RF positivity	32 (78.05%)^#^	25 (60.98%)^#^	0.09
ACPA positivity	30 (78.95%)^§^	32 (86.49%)^†^	0.39
DAS28	4.86 (1.50)	4.80 (1.77)	0.86
Presence of erosions in hand and/or feet X-ray	19 (63.33%)^▲^	27 (77.14%)^▼^	0.22

Data is presented as mean (standard deviation) for continuous variables and number (percentage) for categorical variables.

# Data available for 41 patients.

§ Data available for 38 patients.

† Data available for 37 patients.

▲ Data available for 30 patients.

▼ Data available for 35 patients. ACPA- anti-citrullinated protein antibodies. DAS28- disease activity score 28. DRA- diabetic RA patients. RF- rheumatoid factor. NDRA- non-diabetic RA patients.

### Ultrasonography

Ultrasonography was performed with GE Logiq 5 Expert with a 7-12 MHz linear array transducer. Atherosclerotic plaques and IMT were assessed according to a protocol described elsewhere.^12^

### Statistical analysis

All statistical analyses were performed with STATISTICA 10.0 (StatSoft). Analyses of differences between the groups were performed with parametric (t-test) or non-parametric (Mann-Whitney U) test, depending on data distribution. Categorical variables were compared with chi-squared test. Pearson correlation analysis and Spearman correlation analysis (for linear and categorical variables, respectively) were used to analyze correlations between clinical and laboratory characteristics and IMT. Multivariate stepwise discriminant analysis was used to characterize factors predictive of the presence of atherosclerosis. A *P* value <0.05 was considered statistically significant.

## RESULTS

All groups were age- and sex-matched (Table-I). The percentages of smokers and BMI were similar in all groups. Hypertension was more prevalent in NDRA and DRA groups than in controls. DRA patients had lower HDL concentration than NDRA patients and controls. Total cholesterol and LDL concentrations were higher in controls than in NDRA and DRA groups. A total risk conferred by classical risk factors (FSS) was significantly higher in DRA than in the other groups (Table-I). No significant differences were found in DAS28, erythrocyte sedimentation rate (ESR), concentration of C-reactive protein (CRP), prevalence of rheumatoid factor (RF) or anti-citrullinated protein antibody (ACPA) positivity and bone erosions between NDRA and DRA groups (Tables-I, Table-II). Plaque was more prevalent in DRA patients than in the other groups. CIMT and femoral IMT (FIMT) were insignificantly higher in NDRA group than in controls. Significant differences in CIMT and FIMT were found between DRA patients and controls. CIMT and FIMT were also higher in DRA group than in NDRA group but only the latter difference was significant (Table-III).

**Table-III:** Presence of atherosclerotic plaque and intima media thickness in study and control groups.

	Controls (n=42)	NDRA (n=42)	DRA (n=42)	P value	Post-hoc analysis
Presence of atherosclerotic plaque in carotid and/or femoral arteries	7 (16.67%)	9 (21.95%)	21 (50.00%)	0.0015	Controls vs NDRA: *P*=0.54
					Controls vs DRA: *P*=0.0012
					NDRA vs DRA: *P*=0.008
Presence of atherosclerotic plaque in carotid arteries	5 (11.90)	6 (14.29%)	16 (38.10%)	0.005	Controls vs NDRA: *P*=0.40
					Controls vs DRA: *P*=0.0056
					NDRA vs DRA: *P*=0.013
Presence of atherosclerotic plaque in femoral arteries	2 (4.76%)	4 (9.52%)	13 (30.95%)	0.0017	Controls vs NDRA: *P*=0.17
					Controls vs DRA: *P*=0.0017
					NDRA vs DRA: *P*=0.014
CIMT, mm	0.710 (0.111)	0.771 (0.166)	0.825 (0.191)	0.005	Controls vs NDRA: *P*=0.08
					Controls vs DRA: *P*=0.001
					NDRA vs DRA: *P*=0.12
FIMT, mm	0.485 (0.096)	0.538 (0.178)	0.635 (0.248)	0.001	Controls vs NDRA: *P*=0.19
					Controls vs DRA: *P*=0.0003
					NDRA vs DRA: *P*=0.019

CIMT- carotid intima media thickness. DRA- diabetic RA patients. FIMT- femoral intima media thickness. NDRA- non-diabetic RA patients. RF- rheumatoid factor.

Classical CV risk factors (age, smoking, hypertension, lipid profile, BMI, FSS) and RA-related factors (disease duration, RF, ACPA, DAS28, ESR, CRP, bone erosions) were assessed and their associations with plaque presence and IMT were analyzed. In controls CIMT and FIMT were associated with age (r^2^=0.23, *P*=0.001 and r^2^=0.24, *P*=0.001, respectively) and hypertension (*P*=0.049 and *P*=0.02, respectively); FIMT was also correlated with FSS (r^2^=0.12, *P=*0.026). In multivariate analysis both, age and hypertension, were significantly associated with FIMT (*P*=0.0026 and *P*=0.048, respectively) but only age was associated with CIMT (*P*=0.003). None of the factors analyzed was associated with plaque in controls (data not shown). In NDRA patients plaque was associated with age (*P=*0.006), CIMT- with hypertension, age, BMI, ESR (*P*=0.049; r^2^=0.22, *P*=0.002; r^2^=0.11, *P*=0.029; r^2^=0.11, *P*=0.03, respectively) and FIMT- with hypertension, age, pack-years of smoking, ESR, CRP, FSS (*P*=0.022; r^2^=0.14, *P*=0.017; r^2^=0.12, *P*=0.023; r^2^=0.17, *P*=0.006, r^2^=0.23, *P*=0.001; r^2^=0.17, *P*=0.01, respectively); in multivariate analysis CIMT was associated with age (*P*=0.02) and FIMT- with age and CRP (*P*=0.02 and *P*=0.001, respectively). DRA patients showed an association of plaque, CIMT and FIMT with age only (*P*=0.002; r^2^=0.31, *P*=0.0001; r^2^=0.26, *P*=0.0006, respectively).

To assess if femoral ultrasonography would add information to that provided by the carotid ultrasonography, the prevalence of increased IMT (defined as IMT>97.5 percentile; 97.5 percentile was calculated for CIMT - 0.900mm - and FIMT - 0.700 mm - in controls) and plaque in carotid versus carotid plus femoral arteries was compared in all groups (Table-IV). The prevalence of increased IMT and plaque was slightly higher in the combined analysis of carotid and femoral arteries than in the analysis of carotid arteries only in all groups but the differences were not significant.

**Table-IV:** Prevalence of increased IMT and plaque in carotid vs. carotid and femoral arteries.

	CIMT>97.5 percentile and/or plaque in common carotid arteries	CIMT>97.5 percentile and/or FIMT>97.5 percentile and/or plaque in common carotid or superficial femoral arteries	P value
Controls	6/42 (14.29%)	8/42 (19.05%)	0.77
NDRA	13/42 (30.95%)	17/42 (40.48%)	0.49
DRA	20/42 (47.62%)	22/42 (52.38%)	0.83
NDRA + DRA	33/84 (39.29%)	39/84 (46.43%)	0.44

CIMT- carotid intima media thickness. DRA- diabetic RA patients. FIMT- femoral intima media thickness. NDRA- non-diabetic RA patients.

To exclude potentially confounding effects of treatment with disease modifying anti-rheumatic drugs (DMARDs), treatment regimens in NDRA and DRA groups were compared (Table-V). The percentages of patients treated with methotrexate and biologics, mean doses of methotrexate and percentages of patients treated continuously with DMARDs were similar in both groups.

**Table-V:** Comparison of treatment regimens in NDRA and DRA groups.

	NDRA(n=42)	DRA(n=42)	P value
Patients taking MTX	39 (92.86%)	39 (92.86%)	1.0
Mean dose of MTX, mg/week	20.60 (3.99)	20.45 (5.96)	0.91
Patients taking biologics	4 (9.52%)	7 (16.67%)	0.33
Continuous treatment with DMARDs	15 (35.71%)	18 (42.86%)	0.5

DMARDs- disease modifying anti-rheumatic drugs. DRA- diabetic RA patients. MTX- methotrexate. NDRA- non-diabetic RA patients.

## DISCUSSION

We compared the prevalence of CV risk factors in controls, NDRA and DRA patients and demonstrated lower HDL concentration in DRA group than in NDRA group and controls. The hypertension was more prevalent in NDRA and DRA groups compared to controls. Total CV risk calculated with FSS was nearly twice as high for DRA patients than for NDRA patients and controls. Such an accumulation of classical CV risk factors in DRA group is not surprising as type 2 DM often develops on the basis of MS.

CIMT, FIMT and plaques were positively associated with age in all groups which confirms that age is a strong risk factor of subclinical atherosclerosis. A correlation between CRP and FIMT in NDRA patients corroborated with the findings of previous studies reporting an association between RA activity, inflammatory markers and subclinical atherosclerosis or increased CV morbidity and mortality.^13^-^16^

Our study showed gradually increasing IMT and the prevalence of plaque across the groups analyzed. Plaque was significantly more prevalent in DRA patients than in NDRA patients and controls. FIMT and CIMT were higher in DRA patients than in controls. FIMT was also higher in DRA than in NDRA patients. These findings suggest that DM substantially increases CV risk in RA patients. As we did not find significant differences in inflammatory markers, RA activity, bone erosion prevalence, RA treatment regimens, RF- and ACPA-positivity, the observed increased IMT and prevalence of plaque can be solely attributed to DM and the associated metabolic abnormalities. Only few studies have assessed the influence of abnormalities in glucose metabolism on atherosclerosis in RA. However, most of them have focused on IR and MS and not on DM. Dessein et al. reported an association between IR and CIMT.^10^ Chung et al. found that MS correlated with coronary artery calcification.^8^ Pamuk et al. observed that Homeostasis Model Assessment-IR (HOMA-IR)was associated with carotid plaque in RA patients.^17^ La Montagna et al. found a correlation between HOMA-IR and CIMT in RA.^18^ Del Rincon et al. reported a higher prevalence of DM in RA patients with evidence of plaque in carotid ultrasound.^16^ Our study was designed to compare markers of subclinical atherosclerosis in diabetic versus non-diabetic RA patients. The results suggest that DM is an independent risk factor for subclinical atherosclerosis in RA.

The data on the territorial distribution of atherosclerosis in RA are conflicting. Protogerou et al. reported that subclinical femoral atherosclerosis in RA is analogous to DM.^19^ On the other hand the CARRÉ Investigation showed the predominance of coronary artery disease in RA as opposed to peripheral arterial disease in type 2 DM.^3^ Similarly, a study by Stamatelopoulos et al. suggests a less pronounced pro-atherosclerotic effect of RA on the femoral arteries in comparison to carotid arteries while studies by Li et al. and Danese et al. showed a significantly higher prevalence of atherosclerosis in the lower extremity arteries than in the carotid arteries in type 2 diabetes.^2^,^11^,^20^ In our study FIMT was significantly increased in DRA patients compared to NDRA patients (0.092mm) while the difference in CIMT was insignificant. On the other hand, we did not observe a higher detection of subclinical atherosclerosis in NDRA and DRA patients by using combined carotid and femoral ultrasonography compared to sole carotid ultrasonography. The results observed in NDRA patients are in agreement with our previous study on a large non-diabetic RA cohort.^12^ The surprising result was that we observed no advantage of combined carotid and femoral ultrasonography in detecting atherosclerosis in DRA group. Former studies suggested a more pronounced pro-atherosclerotic effect of DM on the femoral arteries.^2^,^11^,^20^ Our study did not confirm this finding in RA population. However, it should be emphasized that our DRA group was relatively small and further studies are needed to confirm our findings.

In conclusion, we found increased subclinical atherosclerosis in diabetic RA patients as compared to non-diabetic RA patients. Our study suggests that a combination of carotid and femoral ultrasonography does not increase the detection of atherosclerosis in RA, irrespectively of the coexistence of DM.
